# MXenes as a versatile platform for reactive surface modification and superior sodium‐ion storages

**DOI:** 10.1002/EXP.20210024

**Published:** 2021-10-30

**Authors:** Jinjin Wang, Cheng‐Feng Du, Yaqing Xue, Xianyi Tan, Jinzhao Kang, Yan Gao, Hong Yu, Qingyu Yan

**Affiliations:** ^1^ State Key Laboratory of Solidification Processing Center of Advanced Lubrication and Seal Materials Northwestern Polytechnical University Xi'an Shaanxi P. R. China; ^2^ School of Materials Science and Engineering Nanyang Technological University Singapore

**Keywords:** energy, MXene, nanohybrids, sodium‐ion storage, surface modify

## Abstract

Owing to the large surface area and adjustable surface properties, the two‐dimensional (2D) MXenes have revealed the great potential in constructing hybrid materials and for Na‐ion storage (SIS). In particular, the facilitated Na‐ion adsorption, intercalation, and migration on MXenes can be achieved by surface modification. Herein, a new surface modification strategy on MXenes, namely, the reactive surface modification (RSM), is focused and illustrated, while the recent advances in the research of SIS performance based on MXenes and their derivatives obtained from the RSM process are briefly summarized as well. In the second section, the intrinsic surface chemistries of MXenes and their surface‐related physicochemical properties are first summarized. Meanwhile, the close relationship between the surface characters and the Na‐ion adsorption, intercalation, and migration on MXenes is emphasized. Following the SIS properties of MXenes, the surface‐induced SIS property variations, and the SIS performance of RSM MXene‐based hybrids are discussed progressively. Finally, the existing challenges and prospects on the RSM MXene‐based hybrids for SIS are proposed.

## Graphical abstract

Given the potential of MXenes towards Na‐ion storage, the recent advances of Na‐ion adsorption, intercalation, and migration on MXenes and their hybrids generated from reactive surface modification are summarized.

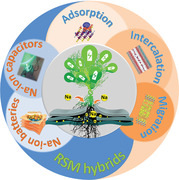

## 1 INTRODUCTION

The continuous growth of the global population and economy dramatically raises the demands for energy supply, which brings about skyrocketing environmental issues.^[^ ^1^, ^2^ ^]^ Upon the urgent application of clean energy harvesting technologies including solar, wind, tidal, etc.,^[^ ^3^, ^4^, ^5^ ^]^ developing large‐scale electrochemical energy storage devices (EESDs) becomes critical for efficiently utilizing these intermittent renewable energies.^[^ ^6^, ^7^, ^8^ ^]^ Among various EESDs, great success has been achieved in lithium‐ion batteries (LIBs) since the 1990s, and have been rapidly developed into a major force in energy storage. Nowadays, LIBs possessing satisfactory energy density and cyclic stability are commonly used in portable devices and electric vehicles.^[^ ^9^, ^10^ ^]^ However, the maldistribution and exhaustion of lithium resources lead to an unsuitable high cost for large‐scale applications.^[^ ^11^, ^12^, ^13^ ^]^ Alternatively, sodium‐ion storage (SIS) devices have attracted wide attention because of the rich sodium resources, similar physicochemical properties of sodium to those of lithium, and the accessibility of sodium.^[^ ^14^, ^15^, ^16^ ^]^ Nevertheless, due to the higher reduction potential of Na^+^/Na and the large ionic radius, the SIS devices still suffer from a low energy density and sluggish kinetics, which hinder their progress to large‐scale commercialization.^[^ ^17^, ^18^ ^]^

Up to now, numerous cathode (layered transition metal oxides,^[^ ^19^, ^20^, ^21^, ^22^, ^23^, ^24^ ^]^ Prussian blue analogs,^[^ ^25^ ^]^ polyanionic compounds,^[^ ^26^, ^27^, ^28^, ^29^, ^30^, ^31^, ^32^ ^]^ and organic materials^[^ ^33^ ^]^) and anode materials (carbonaceous materials,^[^ ^34^, ^35^, ^36^, ^37^, ^38^ ^]^ transition metal oxides and sulfides,^[^ ^39^, ^40^, ^41^, ^42^, ^43^, ^44^, ^45^ ^]^ and alloying^[^ ^46^, ^47^, ^48^ ^]^) have been investigated for SIS. Unfortunately, most of them suffer from inferior performance. For example, carbonaceous materials feature low cost and high security, but the low initial coulombic efficiency and poor cyclic stability are intractable.^[^ ^37^ ^]^ Transition metal oxides and sulfides, though with high theoretical capacities, usually undergo very complicated multiphase transition reactions during the electrochemical process, which lead to the collapse of the host structure and therefore present poor cycling performance for SIS.^[^ ^49^ ^]^ Similarly, the alloy materials possess high conductivity and theoretical capacities, while the volumetric expansion during sodiation becomes even larger, which leads to faster material pulverization.^[^ ^36^ ^]^ In addition, the polyanionic framework compounds are constructed through rigid covalent bonds, present good structural stability for Na‐ion accommodation. However, the inherent poor electrical conductivity of the polyanionic skeleton leads to much slower kinetics for SIS applications.^[^ ^50^ ^]^ Therefore, composite electrode materials with considerable specific capacity and excellent cycle life are intensively pursued.

**FIGURE 1:**
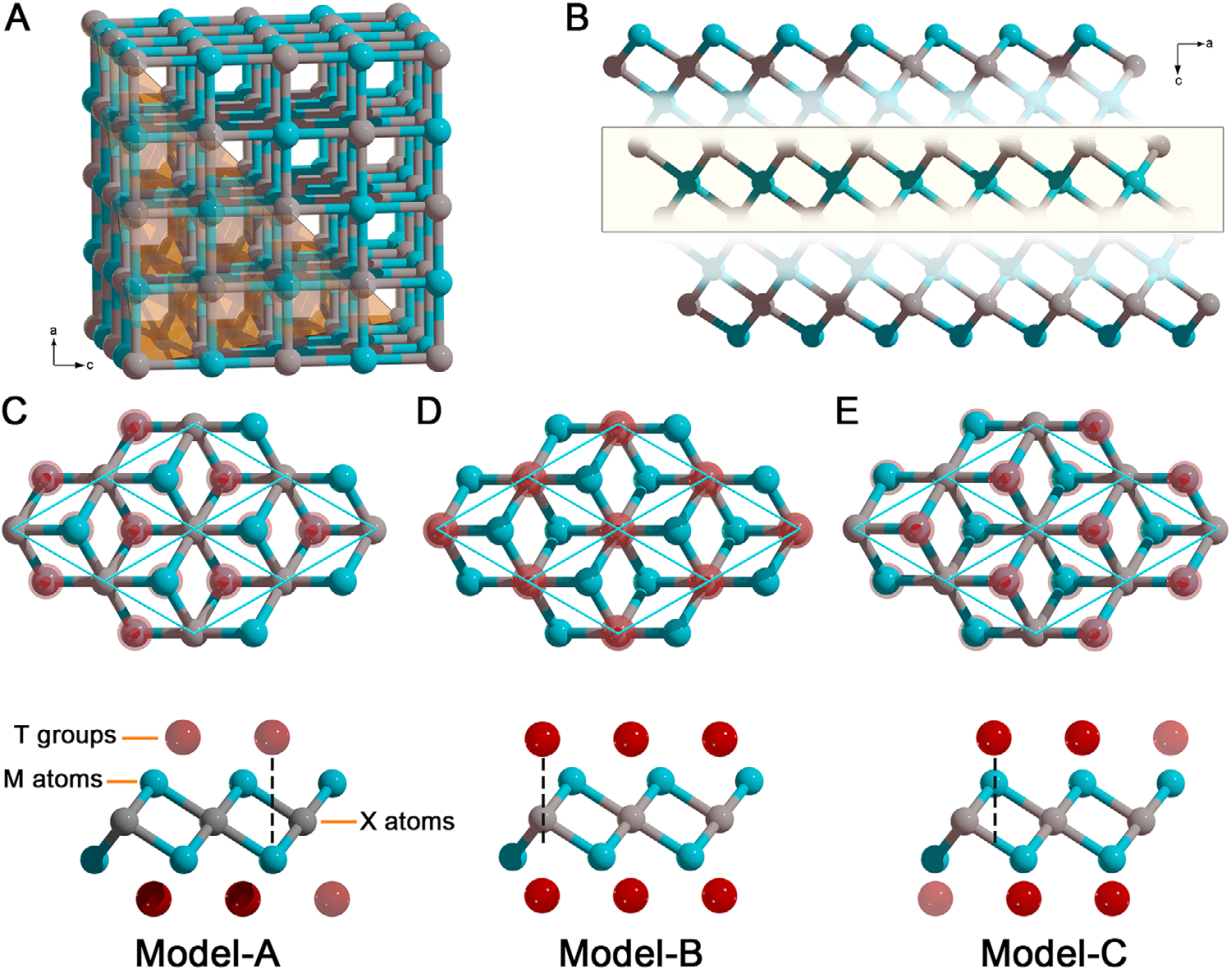
Schematic illustration of (A) the *fcc* lattice of MX and (B) the structure of M*_n_*_+1_X*_n_* MXene layer. The highlight in (A) and (B) shows a MX_2_ layer, respectively. (C–E), The top view (top) and side view (bottom) of three possible positions for T groups on M_2_C type MXene surface with Model‐A, Model‐B, and Model‐C configuration, respectively

MXenes, the transition metal carbides or nitrides which feature two‐dimensional (2D) structures, have gained extensive investigation and rapid development in the past decade.^[^ ^51^ ^]^ Nowadays, more than 200 kinds of stable MXenes have been predicted or even prepared in the lab.^[^ ^52^ ^]^ From a structural perspective, the MXenes represented by a general skeleton formula of M*_n_*_+1_X*_n_* can be considered as the derivatives of MAX phase ceramics by removing the A atoms (M: early transition metals; A: V‐A or VI‐A elements; X: C or N; *n* = 1 to 5).^[^ ^53^, ^54^, ^55^, ^56^, ^57^, ^58^ ^]^ As shown in Figure 1A,B, the ideal MXene monolayer reveals a typical hexagonal lattice with exposed surface metal layer, which is in accordance with the arrangement of *fcc* metal carbides’ lattice along (111) plane. Unsurprisingly, the removal of A atoms results in vast dangling bonds on bare MXene layer, making it an energetically unstable system. Therefore, the surface terminal groups (T groups, ‐F, ‐OH, ‐O) are introduced during the etching process.^[^ ^59^ ^]^ On account of the superior conductivity and large specific area, MXenes have aroused intensive concern in the field of EESDs since their development.^[^ ^60^, ^61^, ^62^ ^]^ Remarkably, on account of the unique surface transition metal layer and the versatile terminal groups, MXenes have shown great capabilities on surface‐involved chemical reactions and have become a versatile platform for hybrid material construction. Recently, a reactive surface modification (RSM) strategy for MXene‐based hybrids was proposed.^[^ ^63^, ^64^ ^]^ Generally, the RSM strategy on MXenes refers to the reaction process which involves at least one species on the MXene sheets as the reactant, while the MXene sheets could still be preserved in the resulting hybrids. Given the *in‐situ* integrated secondary phase, faster charge transfer and reaction kinetics are expected for the RSM MXene‐based hybrids, which have shown superior electrochemical performance than the isolated component.

Until now, there have been some reviews on MXene‐based materials for SIS applications.^[^ ^65^, ^66^, ^67^, ^68^ ^]^ However, most of them only emphasized the synthetic methods, composite structures, and electrochemical performance of the material, while a rare glance has been laid on the intrinsic synergistic mechanism of each component. Meanwhile, considering the RSM MXene‐based hybrids have been widely applied for SIS devices, a systematic summary of these materials is urgent for a deeper understanding of the origin of the SIS performance enhancement. Therefore, a brief summary of the RSM MXene‐based hybrids on SIS applications is provided here, offering the panorama of this advanced material system. In the beginning, the fundamentals of MXenes and their intrinsic surface chemistries are illustrated, followed by the mechanistic studies of surface‐dependent sodium‐ion (Na‐ion) adsorption, intercalation, and migration behaviors. In the third part, recent works on RSM MXene‐hybrids for SIS applications are briefly summarized, which are classified according to the SIS mechanism. At last, the critical challenges of the RSM MXene‐hybrids for SIS at present are briefly discussed, as well as their future developments.

## 2 THE ADSORPTION, INTERCALATION, AND MIGRATION OF SODIUM‐ION ON MXENES

### 2.1 The intrinsic surface chemistries of MXene

In early 2012, the electronic structure variation of Ti‐based MXenes from the parent MAX phases was studied by density functional theory (DFT) simulations, which showed 2.5 to 4.5 times higher density of states than that of the pristine counterpart near the Femi level, and can be related to the delocalized Ti 3*d* states after exfoliation.^[^ ^69^ ^]^ As confirmed by numerous DFT simulations and experimental observations in recent years, the T groups were found to be necessary for a stable MXene layer, and the energetically most favorable positions of T groups were situated above the hollow sites in the center of the three neighboring C atoms (Model‐A, Figure 1C), above the hollow sites on the top of neighboring C atom (Model‐B, Figure 1D), or the mixed configuration of them.^[^ ^70^, ^71^ ^]^ While for the position of T groups that were located on top of neighboring M atoms (Model‐C, Figure 1E), the configuration was energetically unstable.^[^ ^72^ ^]^ Depending on the synthetic condition, T groups can be varied from ‐F, ‐OH, ‐O to ‐Cl, ‐Br, ‐NH, and even ‐S, ‐Se, ‐C.^[^ ^73^ ^]^ A recent DFT simulation of phonon frequency and molecular dynamics have shown that some of the M_2_CS_2_ MXene species can be dynamically stable up to 1000 K.^[^ ^74^ ^]^ However, the ‐O was still the experimentally most stable species among the multifarious terminal groups for most of the MXenes, whereas the Sc‐based MXenes with the preferential ‐F termination are exceptive.^[^ ^75^ ^]^ An X‐ray photoelectron spectroscopy (XPS) study has been applied to detect the evolution of T groups over long‐term exposure in air, in which the ‐F and ‐OH groups were found to be gradually substituted by ‐O.^[^ ^76^ ^]^ Note that even with a small overpotential of about 100 mV (*vs*. open circuit potential, OCP), the oxidation of MXenes can be triggered.^[^ ^77^ ^]^

On the other hand, both the composition and arrangement of M atoms and the surface T groups have a dramatic impact on the electronic structure of MXene layers, especially on the M*_n_*_+1_X*_n_* T*_x_* MXenes that have a lower thickness (e.g., *n* ≤ 2).^[^ ^78^, ^79^ ^]^ Further increasing the thickness (factor *n*), the electronic structure of the corresponding MXene nanosheets approaches that of the bulk metal carbides. Generally, the *s* and *p* orbitals from T groups can be hybridized with the *d* orbital from surface metal atoms, creating new hybrid states near the Femi level, which are closely tied with the variation of the bandgap, work function, carrier mobility, and other physicochemical properties.^[^ ^78^, ^80^, ^81^, ^82^, ^83^ ^]^ According to Khazaei *et al*., the charged state of surface T groups will also affect the geometrical configuration of the MXene species.^[^ ^70^, ^81^ ^]^ For the ‐F, ‐Cl, ‐Br, and ‐OH groups, only one electron was involved in the filled electronic shells (Model‐A, Figure 1a). While for ‐O and ‐S groups, the higher electron demand made them favorable to adsorb with Model‐B configuration (Figure 1b).

Additionally, the surface T groups are also related to the mechanical properties of MXenes. As exampled by the Sc_2_CT*_x_* MXene, Zhang *et al*. showed the relation between surface T groups and the deformation stability of MXene nanotubes.^[^ ^84^ ^]^ Further studies also confirmed that the ‐O terminated Sc_2_CO_2_ MXene presented smaller lattice parameters and layer thicknesses than the Sc_2_CF_2_ and Sc_2_C(OH)_2_ species, and thus a stronger mechanical strength was predicted in the Sc_2_CO_2_.^[^ ^85^ ^]^

### 2.2 The surface‐dependent sodium‐ion adsorption on MXene

As mentioned above, the surface T groups have a dramatic effect on the physicochemical properties of MXene, which are highly dependent on the etching, exfoliation, storage, and post‐treatment conditions. The surface T groups are all electronegative species, which means that during the first step of cation adsorption, the electrostatic interaction plays a critical role. Meanwhile, in the case of Li‐ion adsorption, some early research has demonstrated a structural transformation of ‐O groups on M_2_CO_2_ type MXenes (M = V, Cr, and Ta) during the continuously increased Li‐ion concentration.^[^ ^86^ ^]^ While for the Sc, Ti, Zr, Nb, and Hf‐based MXenes, the structural transformation of ‐O groups did not happen. On the other hand, the adsorption of Li atom on M_2_CO_2_ type MXenes revealed multilayered characteristics, which would further relate to their low Li‐ion diffusion barriers and high capacities.^[^ ^87^ ^]^

**FIGURE 2:**
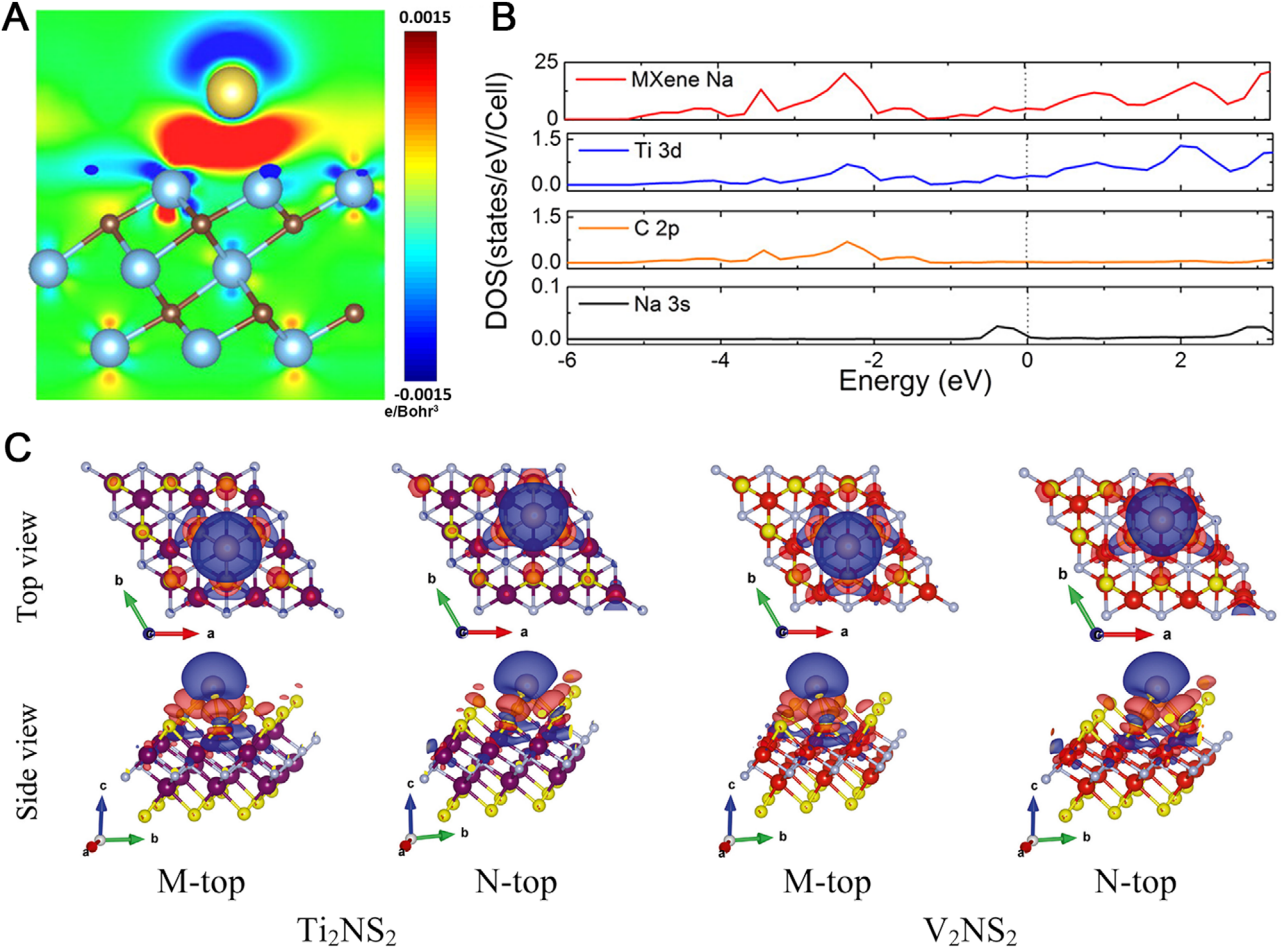
(A) Bonding charge density difference after Na atom adsorbed on Ti_3_C_2_ surface with Model‐A configuration. The electron accumulation and depletion were denoted by red and blue, respectively. (B) Total DOS and PDOS of Ti 3*d*, C 2*p*, and Na 3*s* orbital of Ti_3_C_2_M_1/9_. Dashed lines indicate the Fermi levels. Reproduced with permission.^[^ ^88^ ^]^ Copyright 2021, American Chemical Society. (C) Charge density difference plots for Na adsorption on Ti_2_NS_2_ and V_2_NS_2_ for two different binding sites (M‐top and N‐top) shown from both top and side views (iso‐surface value is 0.001 e Å^−3^). Reproduced with permission.^[^ ^91^ ^]^ Copyright 2019, Elsevier Ltd

For Na‐ion adsorption, the case is quite similar. As predicted by Er *et al*. through DFT simulations,^[^ ^88^ ^]^ the adsorption energy (*E*_ad_) of Na‐ion on bare Ti_3_C_2_ decreased as the coverage increased, which was related to the effective ionic radii of Na‐ion. The coulombic repulsion between Na‐ions was enhanced with respect to their large effective ionic radii, thus lowering the coverage. On the other hand, the adsorbed Na atoms are playing the role of electron provider, in which a part of electrons would transfer from Na atoms to Ti_3_C_2_ MXene. With the Na adsorption, significant *s*‐*d* hybridization was observed between Ti 3*d* orbital and Na 3*s* orbital, demonstrating the strong binding of Na atoms on Ti_3_C_2_ (Figure 2A,B). In consideration of the high theoretical Na capacity of up to 351.8 mAh g^−1^ on Ti_3_C_2_ MXene, stable multilayer adsorption of Na atoms on Ti_3_C_2_ and Ti_3_C_2_O_2_ was predicted. While in the case of Ti_3_C_2_F_2_, the Na showed single‐layered adsorption behavior.^[^ ^89^ ^]^ The Na adsorption on ‐S terminated MXenes (Ti_3_C_2_S_2_, Ti_2_NS_2_, and V_2_NS_2_) were also investigated,^[^ ^90^, ^91^ ^]^ which presented comparable *E*_ad_ of −2.11 eV on Ti_3_C_2_S_2_ with respect to that on Ti_3_C_2_O_2_ (−2.32 eV). For the nitride‐based MXene, the charge transfer from Na to ‐S group was varied as well. As illustrated in Figure 2C, the strong electron sufficient region was observed in both Ti_2_NS_2_ and V_2_NS_2,_ which implying the surface bonding formed by electrons donated from adsorbed Na atoms. When the T groups were replaced by ‐C, the Ti_3_C_4_ monolayer presented a stable surface structure consisting of C_2_ dimers instead of individual C atoms.^[^ ^92^ ^]^ However, the *E*_ad_ of Na on the Ti_3_C_4_ was calculated to be −0.90 eV per atom, which was energetically more unstable than on bare Ti_3_C_2_ (−0.95 eV) and Ti_3_C_2_O_2_ (−2.318 eV).^[^ ^89^ ^]^

In another system based on the Nb_2_C, bare carbide skeleton, and the ‐F and ‐OH terminated analogs were first studied.^[^ ^93^ ^]^ In both cases of Nb_2_CF_2_ and Nb_2_C(OH)_2_, the preferred configuration was Model‐B that is shown in Figure 1B. Interestingly, the bare Nb_2_C seemed more suitable for homogeneous atom adsorption, whereas no Na atoms were adsorbed on the surface of Nb_2_C(OH)_2_. This can be interpreted by the charge transfer from Na to the bare Nb_2_C layer, for which the negative T groups were unfavored. Meanwhile, although the ‐O terminated MXenes were expected to be conducive to EESDs, the authors did not take Nb_2_CO_2_ into consideration. Whereas the Nb_2_CO_2_ reported by Yang *et al*. tends to adopt a stable configuration of Model‐A (Figure 1a) and monolayered Na adsorption according to the DFT results.^[^ ^94^ ^]^ Similar in Sc, V, and Zr‐based MXene systems,^[^ ^95^, ^96^, ^97^ ^]^ the charge transfer between adsorbed Na atoms and the MXene layer were predicted as well, which further proved the variation mechanism of *E*_ad_ corresponding to the electrostatic attraction and charge transfer process.

### 2.3 The surface‐dependent sodium‐ion intercalation on MXene

In 2013, Lukatskaya *et al*. first reported the multiple‐cation intercalation phenomena within Ti_3_C_2_T*_x_* MXene.^[^ ^77^ ^]^ The intercalation behavior of various cations with monovalent (Li^+^ Na^+^, K^+^, and NH_4_^+^), divalent (Mg^2+^), and trivalent (Al^3+^) was expected according to the far exceeding value than that of pure double‐layer capacitances (*C*_dl_) in Ti_3_C_2_T_x_. Furthermore, via DFT simulation, Eames and Islam evaluated the effects of intercalated cations on M_2_X‐type MXenes in detail.^[^ ^52^, ^98^ ^]^ The calculated lattice parameters of intercalated MXene species clearly showed that the cations, the type of transition metals, and the surface T groups on MXenes would all significantly affect the intercalating behaviors.

When turning to the surface chemistries of MXenes, generally, the intercalation behaviors were related to the delocalized electron density from intercalated cations to the MXene surface, resulting in the reduction of the MXene surface.^[^ ^99^ ^]^ For example, the ‐O terminated MXene might accommodate more electrons, thus showed higher cell voltage for Li intercalation than that of the bare MXene or ‐F and ‐OH terminated ones. In 2017, Yang *et al*. explored the intercalation mechanism of Na‐ion in V_2_CT*_x_* MXene via X‐ray absorption near edge structure (XANES).^[^ ^100^ ^]^ During the Na‐ion intercalation/deintercalation, the redox processes of V were observed, which confirmed the charge transfer between the intercalated Na‐ion and MXene surface, and thus the origin of extra electrochemical charge storage capacity.

**FIGURE 3:**
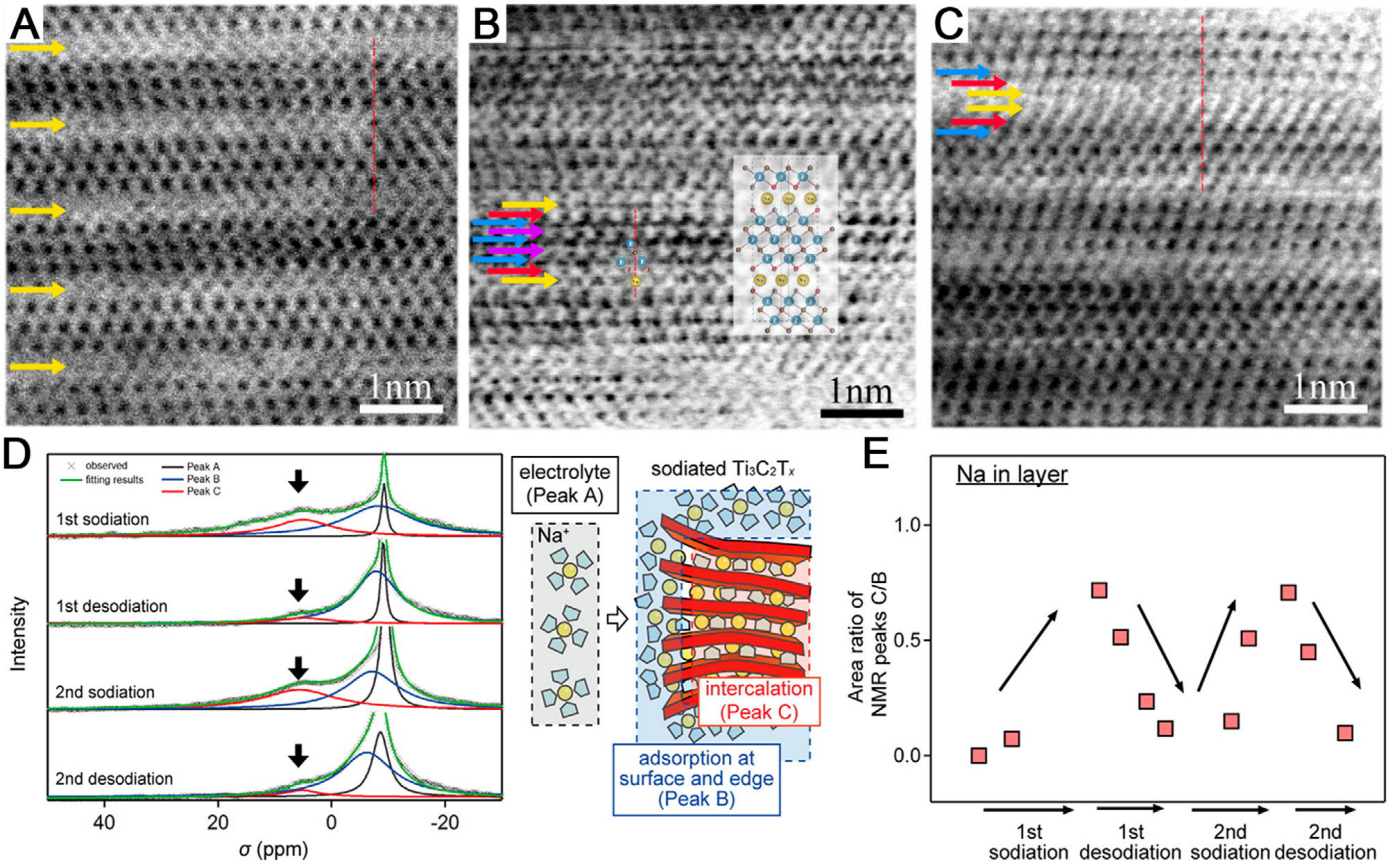
The ABF images of Na intercalation in Ti_3_C_2_T*_x_* under a cutoff potential of (A) 0.5 V and (B) 0.0 V. Reproduced with permission.^[^ ^101^ ^]^ Copyright 2015, American Chemical Society. (D) ^23^Na MAS NMR spectra for the initial two cycles of Na intercalates in Ti_3_C_2_T*_x_*. (E) The fluctuation of intercalated Na was estimated from the NMR peak area ratios of C/B. Reproduced with permission.^[^ ^59^ ^]^ Copyright 2016, American Chemical Society

The intercalation of Na‐ions in the multilayered Ti_3_C_2_T*_x_* MXene was observed firsthand by aberration‐corrected scanning transmission electron microscope (STEM) in 2015.^[^ ^101^ ^]^ As shown in Figures 3A–C, from the annular bright‐field (ABF) images, the intercalation started from the surface of multilayered Ti_3_C_2_T*_x_* MXene, and finally diffused into the bulk. It is worth noting that the favorable position for intercalated Na atoms was located at the top projection site of C atoms, and after intercalation, the shift of T groups was observed. For a fully intercalated sample, double layers of intercalated Na atoms were observed, which were also in accordance with the DFT predictions.^[^ ^102^ ^]^

Based on further studies, the accommodation of Na‐ion in Ti_3_C_2_T*_x_* MXene was dependent on the distension of interlayer spacing during the first sodiation step, in which the solvent molecules swelling and trapped Na‐ions pillaring would happen.^[^ ^59^ ^]^ More importantly, the repulsive/attractive forces between Na‐ion and surface T groups played a critical role in the final interlayer distance. The same phenomenon was also observed in Ti_2_CT*_x_* MXene: the interlayer distance increased by more than 30% during the first sodiation step; while for the following steady Na‐ion intercalation/deintercalation process, little impact was observed on the interlayer distance.^[^ ^54^ ^]^ As proposed in other 2D materials which possess intercalating ion storage mechanism, the ion intercalation is usually accompanied by solvent molecules, e.g., the solvation of Li‐ion and its intercalation in graphite.^[^ ^103^ ^]^ Regarding the case of Na‐ion and MXenes, the ^23^Na magic angle spinning nuclear magnetic resonance (MAS NMR) spectroscopy was applied to explore the intercalation mechanism by distinguishing the adsorbed solvated Na‐ions from the intercalated desolvated Na‐ions in a non‐aqueous system.^[^ ^59^ ^]^ As illustrated in Figure 3D, the NMR peak position and shape of solvated Na‐ions in the electrolyte, solvated Na‐ions adsorbed on Ti_3_C_2_T*_x_* MXene surface, and the intercalated desolvated Na‐ions presented significant differences. During sodiation/desodiation, the amount of intercalated desolvated Na‐ions varied periodically as reflected in the ^23^Na NMR spectra (Figure 3E), suggesting the predominant contribution of desolvated Na‐ion intercalation to the SIS mechanism.

### 2.4 The surface‐dependent sodium‐ion migration on MXene

**FIGURE 4:**
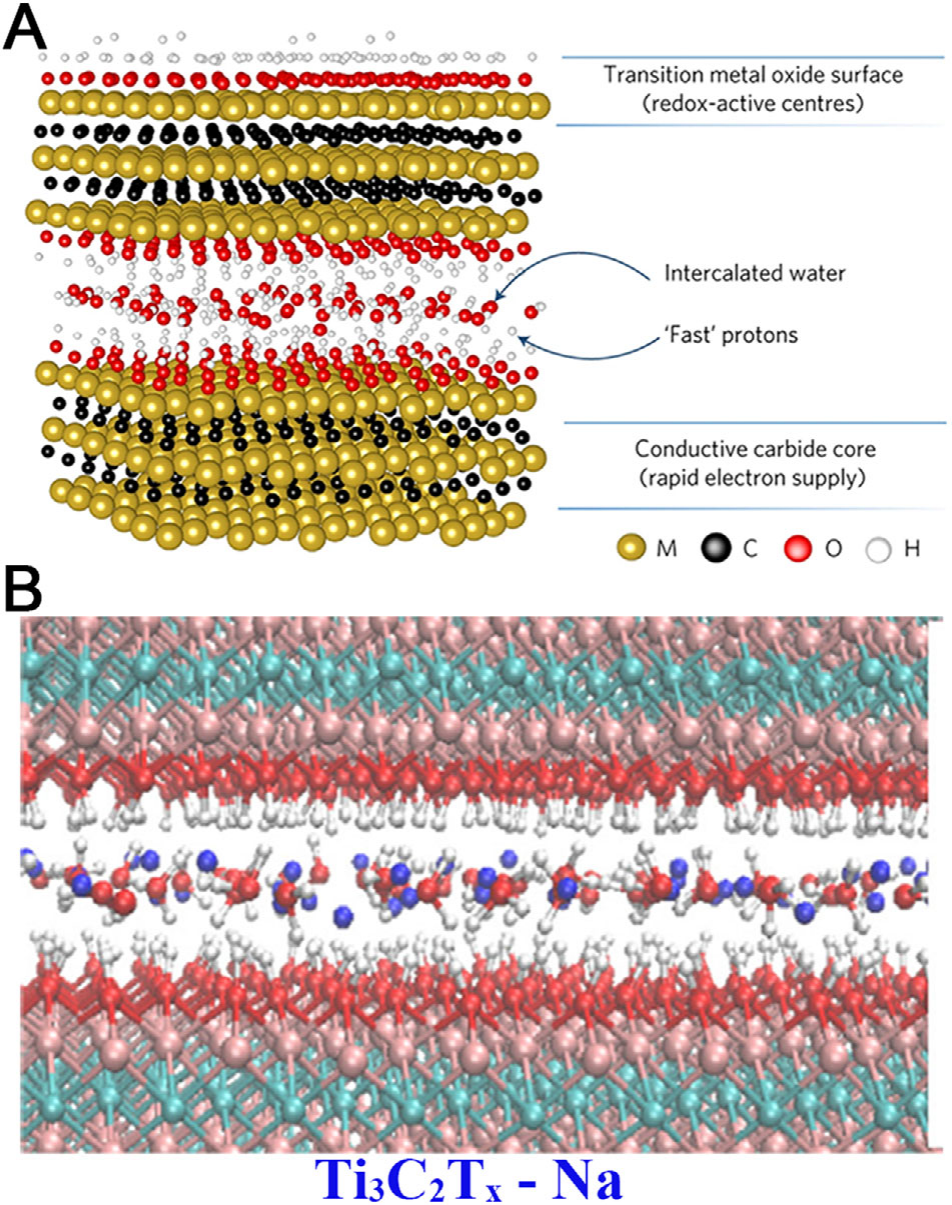
(A) Schematic illustration of water intercalated Ti_3_C_2_T*_x_* MXene. Reproduced with permission.^[^ ^110^ ^]^ Copyright 2017, Macmillan Publishers Limited, part of Springer Nature. (B) The snapshot of Na‐ions intercalated Ti_3_C_2_T*_x_* at 300 K from the ReaxFF molecular‐dynamics simulation. White, red, pink, cyan, and blue atoms represent H, O, Ti, C, and Na, respectively. Reproduced with permission.^[^ ^109^ ^]^ Copyright 2017, American Physical Society

With a volumetric capacitance as high as up to 900 F cm^−3^ in acidic conditions and an excellent rate performance, the diffusion behavior of cations on MXene has received worldwide attention as well.^[^ ^104^ ^]^ Generally, the proton storage and fast transportation in the interlayered space of MXene can be attributed to the “hopping” between neighboring intercalated water molecules and oxygen‐containing T groups on MXene sheets (Figure 4A).^[^ ^105^ ^]^ Confirmed by various techniques, plenty of water molecules were retained in the interlayer space of fresh MXene sheets, and bonded on the MXene surface as well.^[^ ^106^ ^]^ In other words, when considering the ion migration on MXene in an aqueous system, the effect of the water “matrix” cannot be neglected (Figure 4B).^[^ ^107^, ^108^, ^109^ ^]^

**FIGURE 5:**
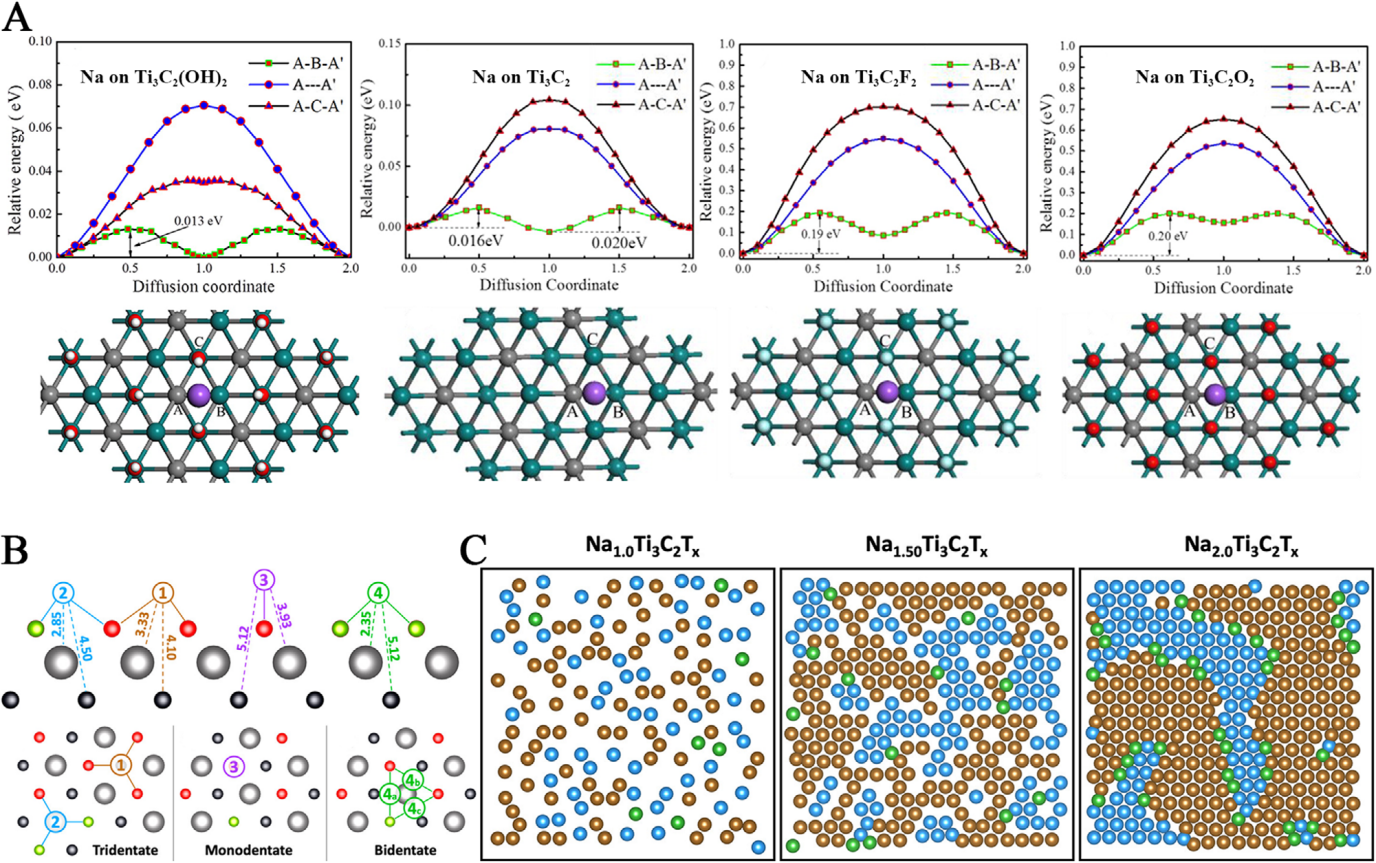
(A) From left to right: energy profiles of different Na atom diffusion paths on ‐OH, bare, ‐F, and ‐O terminated Ti_3_C_2_ MXenes and the corresponding structure of transition states. Reproduced with permission.^[^ ^89^ ^]^ Copyright 2021, American Chemical Society. (B) Side and top views of four Na‐adsorption sites on Ti_3_C_2_T*_x_* surfaces. (C) CMD simulated short‐range ordered surface‐domain structure of sodiated Ti_3_C_2_T*_x_*. The colors of sodiation site follow the descriptions in (B). Reproduced with permission.^[^ ^112^ ^]^ Copyright 2021, American Chemical Society

In a non‐aqueous system, the Na‐ion adsorbed on bare or terminated MXene surfaces are usually adopted for theoretical modeling, in which the diffusion energy barrier (*E*_diff_) of the minimum energy path is commonly taken as the criterion for describing the Na‐ion migration behaviors.^[^ ^89^, ^97^, ^111^ ^]^ As shown in Figure 5A, the *E*_diff_ changed along with the surface of MXenes varying from bare transition metal layer to different T groups. Therefore, possible diffusion paths and corresponding *E*_diff_ of a single Na atom on Ti_3_C_2_‐based MXene were evaluated. For the most probable A‐B‐A’ path, the ‐OH terminated Ti_3_C_2_ showed a significantly reduced *E*_diff_ (0.013 eV) as compared to that of Ti_3_C_2_F_2_ and Ti_3_C_2_O_2_ (0.19 and 0.20 eV, respectively). The higher *E*_diff_ on ‐O/F terminated Ti_3_C_2_ were ascribed to the stronger interaction between the negative terminal groups and Na‐ion, which agreed with the surface‐dependency of *E*_ad_ on MXenes.^[^ ^89^, ^90^ ^]^

In addition to the *E*_ad_ of Na‐ion on a single site, the intercalation environment also affects the *E*_diff_. As exampled by the Ti_3_C_2_‐based MXene; with the interlayer distance of 70 Å, the *E*_diff_ of Na‐ion on bare, ‐F, ‐O, and ‐OH terminated Ti_3_C_2_ MXenes were 0.02, 0.19, 0.20, and 0.013 eV, respectively.^[^ ^89^ ^]^ With a further decrease in interlayer distance from 15 to 10 Å, the Ti_3_C_2_ presented an increased *E*_diff_ from 0.03 to 0.096 eV, respectively; and the Ti_3_C_2_O_2_ revealed an *E*_diff_ of about 0.22 and 0.30 eV with an interlayer distance of 23 and 15 Å, respectively.^[^ ^88^, ^90^, ^102^ ^]^ Obviously, with the narrower interlayer distance between the adjacent MXene layers, the stronger attraction was imposed on the Na‐ion with the higher *E*_diff_ for ion diffusion. Also, it was found that the lattice mismatch between the MXene surface and the adsorbed Na atom layer would significantly affect the energy barrier for Na diffusion. As discussed in Section 2.2, the Ti_3_C_2_S_2_ MXene presented a small variation of *E*_ad_ (< 10%) when compared to that of Ti_3_C_2_O_2_. However, the *E*_diff_ of Na‐ion on Ti_3_C_2_S_2_ was only half of that on Ti_3_C_2_O_2_,^[^ ^90^ ^]^ which was then attributed to the higher similarity in lattice constant of Ti_3_C_2_S_2_ and the optimal Na layers and their higher charge density difference value.

As aforementioned, the behaviors of Na adsorption may be dependent on the concentration of Na‐ions. Similarly, the migration of Na‐ions on the MXene surface also showed a significant correlation with Na‐ion concentration. Brady *et al*., recently drew the patterns of Na‐ion adsorption on Ti_3_C_2_T_x_ MXene with a progressively increasing amount of Na‐ion concentration via classical molecular dynamics (CMD) simulation.^[^ ^112^ ^]^ Surprisingly, the chemical correlations between the ‐O/F terminal groups and Na‐ion created a 2D domain pattern of sodium distributions (Figures 5B,C). With a high pre‐intercalated amount of Na, the Na_2.0_Ti_3_C_2_T*_x_* presented excellent rate capability and high specific energy (249 Wh kg^−1^), which demonstrated the strong impact of the 2D Na‐ion surface‐domains on the migration behavior of Na‐ion.

**FIGURE 6:**
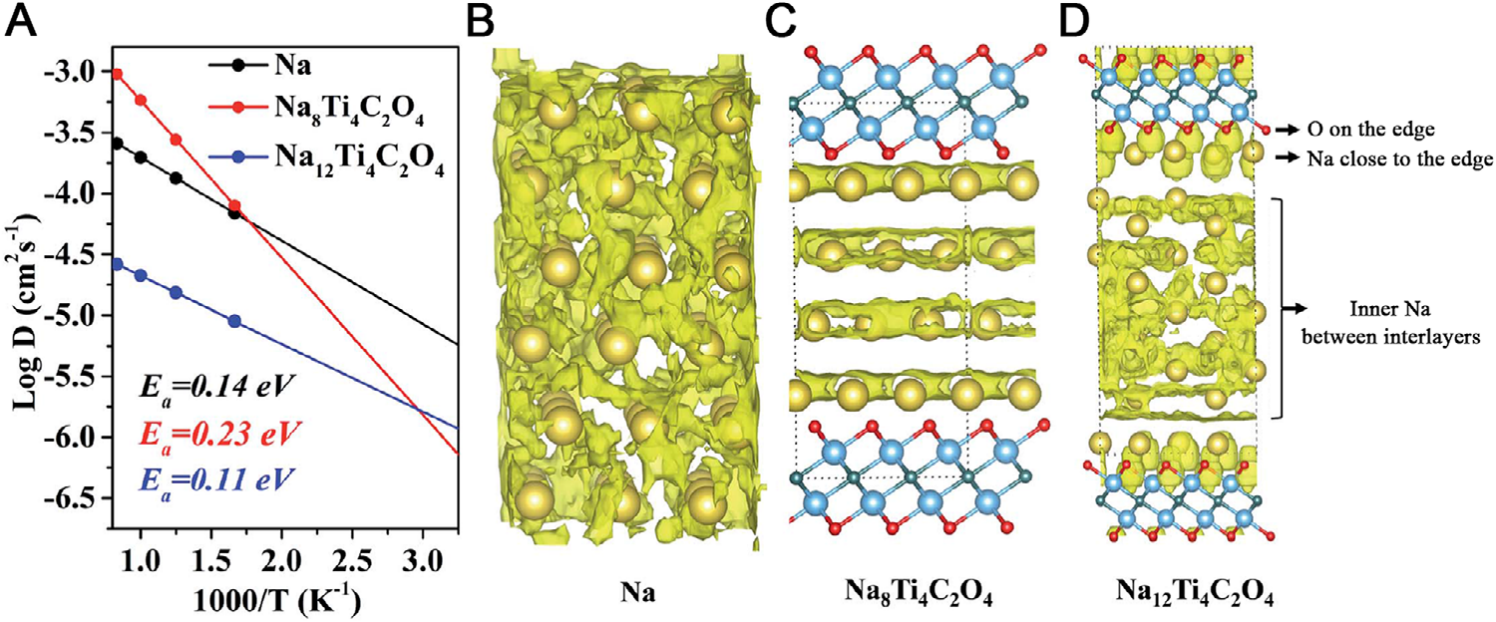
(A) AIMD simulated diffusion coefficients for Na atom in bulk Na, Na_8_Ti_4_C_2_O_4_, and Na_12_Ti_4_C_2_O_4_ from 600 to 1200 K. (B–D) Illustrations of the Na atom trajectories in bulk Na, Na_8_Ti_4_C_2_O_4_, and Na_12_Ti_4_C_2_O_4_ at 1000 K, respectively. Reproduced with permission.^[^ ^113^ ^]^ Copyright 2020, The Royal Society of Chemistry

With a further increase in the amount of intercalated Na‐ion in the interlayer space of MXene sheets, a screening effect of near‐edge Na‐ion was observed according to the Ab initio molecular dynamics (AIMD) simulations, which might result in a bulk‐like Na diffusion behavior for the inner Na atoms (Figure 6A).^[^ ^113^ ^]^ As shown in Figure 6B, the movement trajectories of Na atoms in metallic Na were connected in the 3D space; while for the Na_8_Ti_4_C_2_O_4_ species, the trajectories of Na atoms followed the 2D model because of the electrostatic attraction from edge O^2−^ groups on MXene (Figure 6C). For Na_12_Ti_4_C_2_O_4_, the screening effect of the near‐edge Na‐ion emerged, which endowed the inner Na atoms between the interlayers with extensively overlapping trajectories (Figure 6D). Consequently, the Na_12_Ti_4_C_2_O_4_ species presented a smaller *E*_diff_ of 0.11 eV than that of metal Na (0.14 eV) and Na_8_Ti_4_C_2_O_4_ species (0.23 eV).

For a more visual representation and comparison of the effects of surface chemical states on SIS performance, the SIS‐related properties of different MXenes predicted by theoretical simulations are briefly summarized in Table 1.

**TABLE 1:** Comparison of the SIS‐related properties of different MXenes through theoretical simulations

MXenes	Terminated group	*E*_ad_ (eV)	Lowest *E*_diff_ (eV)	Average OCV (*vs*. Na/ Na^+^, V)	Specific capacity (mAh g^−1^)	Refs.
Ti_3_C_2_	Bare	−0.95	0.100	0.14	351.8	^[^ ^88^ ^]^
Ti_3_C_2_	Bare	/	0.020	/	413.0	^[^ ^89^ ^]^
Ti_3_C_2_O_2_	−O	/	0.200	/	367.7	
Ti_3_C_2_F_2_	−F	/	0.190	/	151.2	
Ti_3_C_2_(OH)_2_	−OH	/	0.013	/	/	
Ti_3_C_2_S_2_	−S	−2.11	0.110	/	463.0	^[^ ^90^ ^]^
Ti_3_C_4_	−C	−0.90	0.350	0.47	560.0	^[^ ^92^ ^]^
Ti_2_C	Bare	−0.79	0.021	/	348.7	^[^ ^114^ ^]^
Ti_2_C_3_	−C	−2.54	0.155	/	301.6	
Ti_2_CO_2_	−O	−1.45	0.059	/	288.6	
Ti_2_CS_2_	−S	−1.26	0.095	/	246.1	
V_Ti_‐Ti_2_CO_2_	−O	−3.85	0.830	/	/	^[^ ^115^ ^]^
TiC_3_	Bare	−0.50	0.180	0.18	1278.0	^[^ ^116^ ^]^
Ti_3_N_2_	Bare	−1.00	0.041	0.51	312.0	^[^ ^44^ ^]^
Ti_3_N_2_F_2_	−F	−0.50	0.180	0.06	85.0	
Ti_3_N_2_O_2_	−O	−2.00	0.181	0.721	258.0	
Ti_3_N_2_(OH)_2_	−OH	−0.10	/	/	/	
Ti_3_C_2_O_2_	−O	−0.202	0.310	/	∼330.0	^[^ ^117^ ^]^
Ti_3_CNO_2_	−O	−0.196	0.123	/	∼330.0	
Ti_3_N_2_O_2_	−O	−0.166	0.040	/	∼330.0	
Ti_2_NS_2_	−S	/	0.090	0.83	84.8	^[^ ^91^ ^]^
V_2_NS_2_	−S	/	0.090	0.53	99.8	
Nb_2_C	Bare	/	0.015	0.27	271.0	^[^ ^93^ ^]^
Nb_2_C	Bare	−0.363	∼0.025	/	219.0	^[^ ^102^ ^]^
Nb_2_CO_2_	−O	−0.665	∼0.180	/	194.0	
Nb_2_C(OH)_2_	−OH	0.282	/	/	/	
Nb_2_CO_2_	−O	/	/	/	288.0	^[^ ^94^ ^]^
Sc_2_C	Bare	/	0.012	0.24	362.0	^[^ ^95^ ^]^
Zr_3_C_2_	Bare	−0.79	0.030	/	/	^[^ ^96^ ^]^
Zr_3_C_2_O_2_	−O	−1.56	0.320	/	326.0	
Zr_2_C	Bare	−0.77	0.030	/	/	
Zr_2_CO_2_	−O	−0.81	0.290	/	474.0	
V_3_C_2_	Bare	−1.24	0.020	/	606.4	^[^ ^97^ ^]^
V_3_C_2_O_2_	−O	−2.73	0.310	/	513.5	
V_2_C	Bare	−0.528	0.010	0.82	470.6	^[^ ^100^ ^]^
V_2_C	Bare	−0.223	/	/	335.0	^[^ ^102^ ^]^
V_2_CO_2_	−O	−0.876	0.150	0.52	367.4	
V_2_CS_2_	−S	−1.26	0.060	0.49	301.1	^[^ ^118^ ^]^
Cr_2_CO_2_	−O	/	0.090	0.26	276.0	^[^ ^94^ ^]^
Mn_2_C	Bare	−0.44	0.022	0.25	443.6	^[^ ^119^ ^]^
Mn_2_CO_2_	−O	/	0.150	0.80	/	
MnC	Bare	−2.83	0.174	/	475.0	^[^ ^120^ ^]^
Mo_2_C	Bare	−1.01	/	0.31	262.9	^[^ ^121^ ^]^
MoC	Bare	−0.89	/	0.80	248.2	
MoC_2_	Bare	−1.76	0.230	0.28	446.9	
Mo_2_CO_2_	−O	/	0.140	0.19	/	^[^ ^94^ ^]^
MoCrC_2_	Bare	−0.28	0.027	0.89	297.9	^[^ ^122^ ^]^
Sr_2_C	Bare	−0.61	0.012	0.24	362.0	^[^ ^95^ ^]^
o‐Sr_2_C_2_	Bare	−0.28	0.050	0.08	777.0	^[^ ^123^ ^]^
o‐Sr_2_N_2_	Bare	−0.75	0.269	0.10	735.0	
Hf_3_C_2_	Bare	−1.91	0.018	0.46	444.9	^[^ ^124^ ^]^
Hf_3_C_2_F_2_	−F	−0.91	0.083	1.60	/	
Hf_3_C_2_FO_2_	−O	−2.93	0.231	0.46	/	
Hf_3_C_2_(OH)_2_	−OH	−0.94	0.013	3.11	/	
Ca_2_C	Bare	−2.83	0.059	0.52	582.0	^[^ ^125^ ^]^

## 3 RSM MXENE‐HYBRIDS FOR SUPERIOR SODIUM‐ION STORAGE

With regards to the strong correlation between the surface chemical states of MXenes and their SIS properties, the ideas can be further extended for understanding the synergistic enhancement on SIS performance of MXene derivatives, namely, the MXene‐based hybrids derived from the RSM process. In the following section, the RSM MXene‐based hybrids for SIS would be systematically discussed.

### 3.1 RSM towards MXene‐based hybrids

Although the MXenes delivered a promising future for SIS in the DFT simulations, their limitations are also conspicuous in reality. First and foremost, due to the high chemically active surfaces, the agglomeration and restacking of MXene flakes are inevitable. Particularly, the interactions between MXene layers not only consist of van der Waals force, but the hydrogen bonding network also exists between the adjacent ‐OH and ‐O/‐F groups.^[^ ^126^ ^]^ Meanwhile, the oxidation tolerance of MXenes decreases with the decreased thickness, which means that the surface quality of obtained MXene nanoflakes usually deviates from the ideal state. Therefore, unsatisfactory electrochemical properties from the few‐layered MXenes are observed. Furthermore, the limited capacity of bare MXenes is still insufficient for real applications.^[^ ^127^, ^128^ ^]^ Given all the above drawbacks of bare MXenes, the MXene‐based hybrids become a more feasible solution. Indeed, benefiting from the highly active surfaces, the MXenes are regarded as a versatile platform for constructing active hybrids in recent years. Remarkably, the RSM strategy that involves part of the surface atoms from MXene sheets as reactants for generating MXene‐based hybrids is proposed.^[^ ^63^, ^129^ ^]^ With the RSM strategy, the obtained MXene‐based hybrids with controllable surface properties and *in‐situ* generated interlayer spacers may deliver good dispersity and enhanced stability, which will open up a new space for advanced SIS devices.

Depending on the reaction condition, the *in‐situ* conversion of surface metal species into metal oxides was firstly realized via simply hydrothermal/solvothermal or annealing processes.^[^ ^130^, ^131^, ^132^, ^133^ ^]^ The strategies are facile, while the extent of reaction can be controlled by a given atmosphere. However, the products are restricted by the metal species that the MXenes contain. Whereafter, inspired by the *in‐situ* extraction of surface transition metal atoms from MXene layers during redox reactions, the alloy nanoparticles^[^ ^63^, ^129^, ^134^ ^]^ or sodium titanate (NaTi_1.5_O_8.3_, Na_0.23_TiO_2_, Na_2_Ti_3_O_7_, and NaTi_8_O_13_/NaTiO_2_)‐based hybrids were obtained under the existence of exogenous reagents following the similar strategy as well.^[^ ^135^, ^136^ ^]^ To obtain the RSM MXene‐based hybrids, direct hydrothermal/solvothermal growth of the secondary materials on MXenes is still the most universal method. Particularly, the oxygen‐rich or alkali condition will result in the replacement of surface ‐F groups by the more active ‐OH groups,^[^ ^137^, ^138^ ^]^ which will further facilitate the formation of secondary phases during the subsequent phase transformation process. For instance, the MXenes could be the metal source for *in‐situ* conversion towards MXene‐based hybrids, where the NaTi_2_(PO_4_)_3_ cubes were grown on Ti_3_C_2_ layers in one step through a hydrothermal route.^[^ ^139^ ^]^ Nevertheless, although the variety of RSM reactions have expanded, the reaction progress has become harder to control. For example, with the alkali treatment on Ti_3_C_2_T*_x_*, the products can either be a hybrid of titanate on MXenes,^[^ ^138^ ^]^ or simply the titanate.^[^ ^140^ ^]^ Therefore, a restriction on the number of exogenous reagents is further adopted, and the RSM process is divided into separate steps.^[^ ^64^, ^141^ ^]^ Exampled by the pre‐assembly methods, a certain amount of exogenous reagents is introduced on MXenes, and the subsequent heat‐treatment is applied to trigger the RSM reaction. In this way, the RSM products can be further extended to alloys, chalcogenides, phosphides, and so on. Meanwhile, the extent of reaction can be fine‐tuned. In 2018, an *in‐situ* conversion of surface Mo atoms into MoS_2_ nanosheets was realized on Mo_2_TiC_2_ MXene by liquid incorporation and post‐sulfidation,^[^ ^142^ ^]^ which showed excellent Li‐ion storage performance.

**FIGURE 7:**
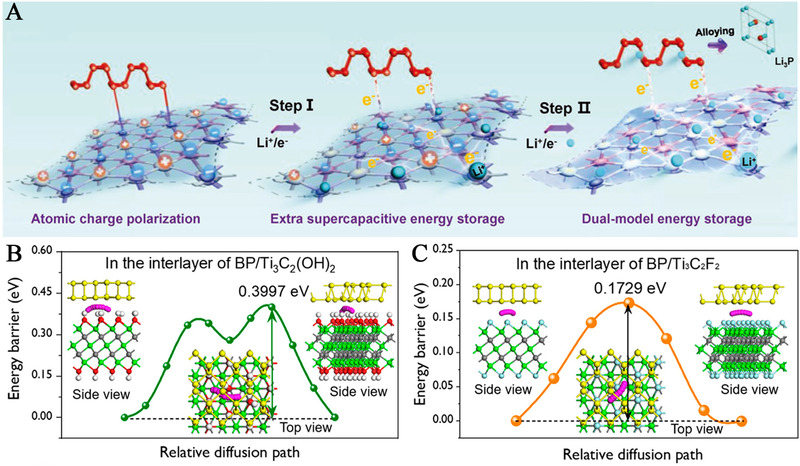
(A) Schematic illustration of the dual‐model energy storage mechanism of the BPQD/TNS electrodes. Reproduced with permission.^[^ ^145^ ^]^ Copyright 2018, Wiley‐VCH. (B,C) Energy barrier and the corresponding diffusion paths (inserts) of a Na atom on the interface of BP/Ti_3_C_2_(OH)_2_ and BP/Ti_3_C_2_F_2_, respectively. Reproduced with permission.^[^ ^146^ ^]^ Copyright 2020, American Chemical Society

It should be particularly noted that although there is a recent boom in constructing MXene‐based heterostructures for SIS, most of the secondary phases are still generated from exogenous reagents, in which the interfaces are connected by weak supramolecular interactions (electrostatic interaction, hydrogen bonding, or van der Waals force).^[^ ^143^, ^144^ ^]^ Meanwhile, the hybrids with secondary phases which covalently bond on MXenes (only by deprotonating and sharing surface O atoms of MXene) should also be discussed individually. For example, through a simple post‐assembly route, the black phosphorus quantum dots (BPQDs), BP nanosheets, and red phosphorus can be coupled with Ti_3_C_2_T*_x_* MXene nanosheets (TNS), in which the two components would connect by the formation of P‐O‐Ti interfacial bonding.^[^ ^145^, ^146^, ^147^ ^]^ With the strong chemical bonding of Ti‐O‐P, the charge transfer was much smoother than that of the interface formed by mere physical mixing, and an additional capacity can be introduced (Figure 7A). Moreover, the surface terminated ‐F groups were found to contribute to a stable solid electrolyte interphase (SEI) film, and enhance the affinities while lowering the diffusion barriers of Na atom (Figure 7B,C).^[^ ^146^ ^]^ Similar interfacial synergy was also observed in MoSe_2_/Ti_3_C_2_T*_x_* hybrid system with Ti‐O‐Mo bonding, and was believed to universally exist in the covalent binding interfaces.^[^ ^148^ ^]^

However, given the scattering effect and the electron localization at the heterogeneous interface constructed by supramolecular interactions, there is a blurred difference between the protogenetic/exogenous secondary phase of post‐assembled covalent hybrids, and there are potential advantages of the coherent interface in interfacial orbital coupling and electronic transportation.^[^ ^149^ ^]^ Here we will only focus on the RSM hybrids that involve a surface atom transferring from MXene layer to the *in‐situ* formed secondary phase.

### 3.2 RSM MXene‐hybrids for advanced sodium‐ion storage

**FIGURE 8:**
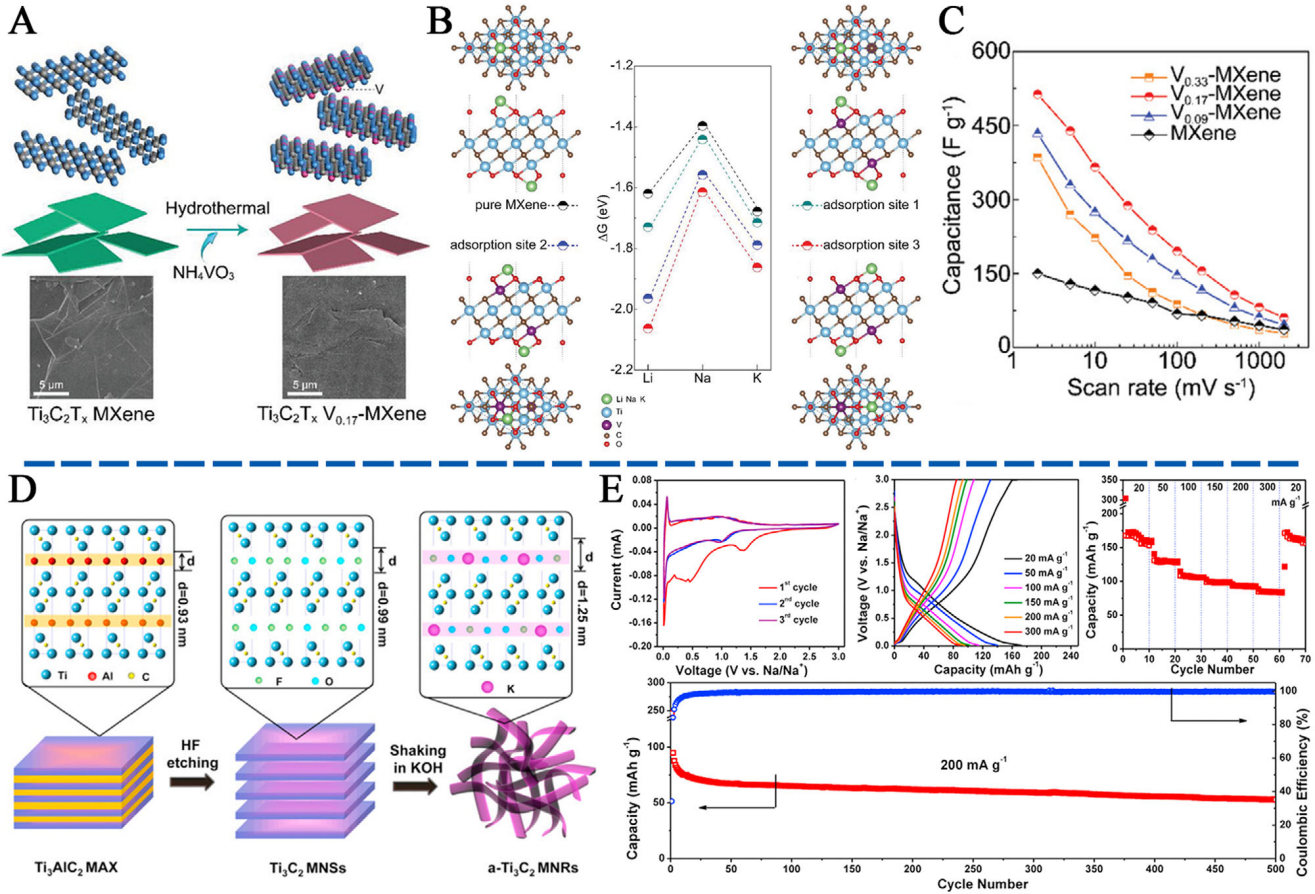
(A) Schematic illustration of the synthesis of V‐doped Ti_3_C_2_T*_x_* MXene nanosheets by the hydrothermal method. (B) The three different alkali metal ion adsorption sites on V‐doped MXene monolayer and the corresponding Gibbs free energy diagram. (C) The relation between scan rate and the mass‐specific capacitance. Reproduced with permission.^[^ ^150^ ^]^ Copyright 2019, Wiley‐VCH. (D) Schematic illustration of the synthesis of a‐Ti_3_C_2_ MNRs. (E) Electrochemical characterizations of a‐Ti_3_C_2_ MNRs for SIBs. Reproduced with permission.^[^ ^138^ ^]^ Copyright 2017, Elsevier Ltd

Gao *et al*. tuned the surface activity of Ti_3_C_2_T*_x_* MXene via the introduction of V as a heteroatom to replace some of the Ti atoms (Figure 8A).^[^ ^150^ ^]^ The surface doped V atom endowed an additional electron transfer from V *d*‐orbital to O *p*‐orbital, hence strengthening the Na‐ion adsorption. As shown in Figure 8B, the Na‐ion adsorption above the vacancy near the doped V atom presented the lowest *E*_ad_, revealing the doping effect of V. With an optimal V content, the V_0.17_‐MXene possessed a high capacitance of 321.7 F g^−1^ at 10 mV s^−1^ for a sodium‐ion capacitor (SIC) in a three‐electrode system with Pt as the counter electrode. While with further increase of V content, the cation diffusion became the dominant limitation, which led to a deteriorated performance (Figure 8C). From another point of view, since the adjustment of surface T groups has been theoretically demonstrated to be an effective way of enhancing Na adsorption, experimental verification should be performed. In a recent work, the Ti_3_C_2_T*_x_* MXene was modified by S atoms through a step‐by‐step annealing process (CT‐S@Ti_3_C_2_‐450).^[^ ^62^ ^]^ Although the authors described the S atoms as “intercalated”, the S‐Ti‐C bonds were detected after the modification. The DFT simulation on Ti_3_C_2_S_2_ revealed that a strong *E*_ad_ of Na atoms (−1.88 eV) was achieved. Even with double‐layered Na adsorption, the *E*_ad_ was still negative. By combining the enhanced *E*_ad_ of Na atoms and enlarged interlayer spacing, the material delivered an improved capacity of 550 mAh g^−1^ at 100 mA g^−1^ and SIS kinetics as an anode for SIC in a half‐cell, as well as excellent capacity retention of 73.3% at 2000 mA g^−1^ over 10,000 cycles in a full‐cell with active carbon as the counter electrode. Meanwhile, Amiri *et al*. modified the surface of Ti_3_C_2_T*_x_* MXene by N atoms via high‐temperature annealing as well.^[^ ^151^ ^]^ The derivative showed good performance on both volumetric/gravimetric electroabsorption capacity and rate performance. Very recently, the surface ‐O groups enriched Ti_3_C_2_T*_x_* (O‐Ti_3_C_2_T*_x_*) was achieved by ascorbic acid (VC) preconditioning, followed by thermal treatment.^[^ ^152^ ^]^ Interestingly, in the study, improved stability of bulk O‐Ti_3_C_2_T*_x_* as anode over the bulk Ti_3_C_2_T*_x_* during high‐rate cycling was observed. Based on the post‐analyses of the cycled materials, the layered structure of Ti_3_C_2_T*_x_* was totally transformed into crystalline nanoparticles (NPs), and the Na_16_Ti_10_O_28_ NPs were also generated *in‐situ* at the edge space. Given the microstructure of Ti_3_C_2_T*_x_* and Na_16_Ti_10_O_28_ NPs, the Na_16_Ti_10_O_28_ NPs were referred to a physical barrier with the collaborative surface Ti‐O groups on Ti_3_C_2_T*_x_* NPs.

As shown by Lian *et al*.,^[^ ^138^ ^]^ the alkali treatment of Ti_3_C_2_T*_x_* MXene (a‐Ti_3_C_2_ MNRs) under the mild and inert conditions not only led to the replacement of surface ‐F to ‐OH groups, but also facilitated the delamination, splitting, and re‐assembly of short MXene nanoribbons (Figure 8D). With the RSM process by KOH, the intercalated K^+^ and repulsive force between O‐terminated groups simultaneously contributed to an enlarged interlayer spacing, which dramatically lowered the electrochemical potential for Na‐ion intercalation. Consequently, when evaluated as anode *vs*. Na plate for sodium‐ion battery (SIB), the reversible Na‐ion intercalation occurred under a low potential of about 1.1 V from the cyclic voltammetry (CV) and Galvanostatic charge/discharge (GCD) curves. Meanwhile, a reversible capacity of 168 mAh g^−1^ was observed in the a‐Ti_3_C_2_ MNRs at 20 mA g^−1^, showing the good potential of MXene derivatives in SIS applications (Figure 8E).

**FIGURE 9:**
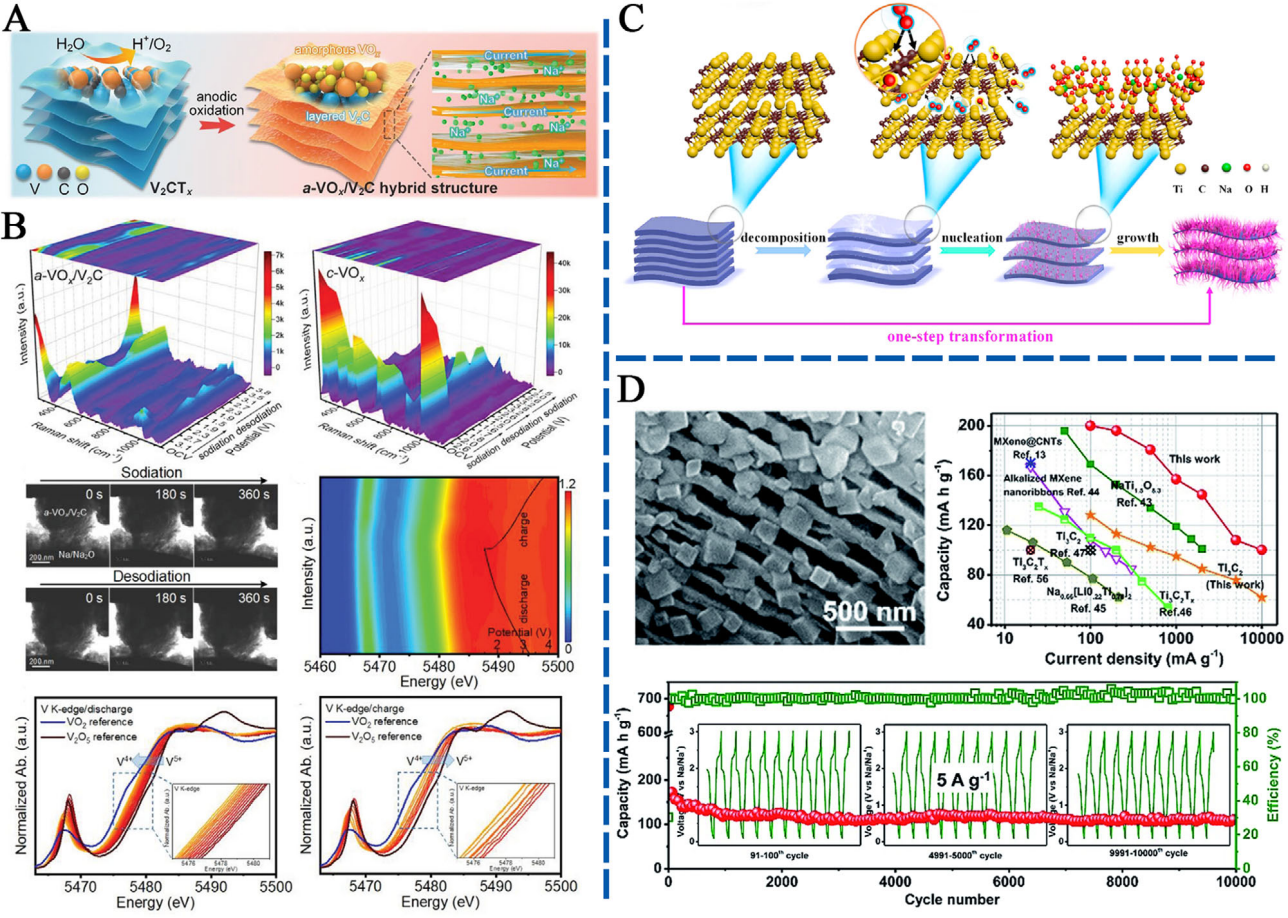
(A) Schematic illustration showing the synthesis of a‐VO*_x_*/V_2_C nanohybrid and, (B) SIS mechanism investigations. Reproduced with permission.^[^ ^154^ ^]^ Copyright 2021, Wiley‐VCH. (C) Schematic representation of the formation mechanism for the sandwich‐like Na_0.23_TiO_2_/Ti_3_C_2_ composite through an *in‐situ* transformation reaction. Reproduced with permission.^[^ ^135^ ^]^ Copyright 2018, Elsevier Ltd. (D) The SEM image and SIS performance of MXene@NTP‐C. Reproduced with permission.^[^ ^139^ ^]^ Copyright 2018, The Royal Society of Chemistry

On one hand, as a typical RSM of MXene, the *in‐situ* oxidation of the surface metal atoms from MXene layers can be realized under O_2_, CO_2_, or even N_2_ atmosphere.^[^ ^130^, ^133^ ^]^ As demonstrated by Wang *et al*.,^[^ ^132^ ^]^ the Ti_3_C_2_T*_x_* MXene was oxidized to TiO_2_ NPs by the pre‐intercalated tetramethylammonium hydroxide (TMAOH) under elevated temperatures. Although no extra pillar agent was introduced, the TiO_2_ NPs attached on the basal and edge planes of Ti_3_C_2_T*_x_* nanosheets can still enlarge the interlayer spacing, which offered a facilitated interaction and diffusion of Na‐ions as well. Recently, a similar TiO_2_/Ti_3_C_2_ nanohybrid was also generated via a controlled hydrothermal oxidation process.^[^ ^153^ ^]^ The accordion‐like TiO_2_/Ti_3_C_2_ nanohybrid anode delivered a stable specific capacity of ∼156 mAh g^−1^ at 50 mA g^−1^ over 500 galvanostatic charge‐discharge cycles in a half‐cell device, and a nearly 100% capacity retention at 200 mA g^−1^ over 500 cycles. The high capacity was ascribed to the joint contribution from Na atom intercalation in Ti_3_C_2_ layers with an enlarged interlayer spacing, and the extra Na‐ion storage in anatase NPs. Recently, the electrochemical anodic oxidation method was applied for RSM MXene‐hybrids as well.^[^ ^154^ ^]^ The V_2_CT*_x_* MXene underwent surface oxidation in Na_2_SO_4_/H_2_SO_4_ aqueous electrolyte, which resulted in an a‐VO*_x_*/V_2_C hybrid (Figure 9A). The detailed SIS mechanism of a‐VO*_x_*/V_2_C hybrid as cathode was detected via various *in‐situ* techniques and compared with bare c‐VO_2_ as well. As shown in Figure 9B, benefiting from the RSM hybrid structure, the a‐VO*_x_*/V_2_C presented a highly reversible Na intercalation/deintercalation behaviors with Na plate as the counter electrode. For the a‐VO*_x_*/V_2_C, an amorphous VO*_x_* layer formed on the V_2_C sheets, which contributed to the reversible (de)intercalation of Na‐ions accompanying an oscillation of valence on V atoms. Meanwhile, the robust V_2_C skeleton provides an efficient conducting network for the facilitated SIS performance. However, for the bare c‐VO_2_, an irreversible valence change of V atoms was observed.

In addition to the Na‐free metal oxides, the Na‐preintercalated phases were also prepared on MXene through the RSM strategy. For example, the delamination and splitting effect of alkaline lye on MXenes was applied in collaboration with oxidation reaction to obtain Na‐contained RSM MXene‐hybrids. At room temperature, the Na_0.23_TiO_2_ nanobelts were grown on Ti_3_C_2_T*_x_* MXene by a simple NaOH solution treatment without inert atmosphere protection (Figure 9C).^[^ ^135^ ^]^ The 3D hybrid architecture, built by 1D ultrathin nanobelts of Na_0.23_TiO_2_ and 2D conductive sheets of MXene, could endow a faster transport process and higher stability for the hybrid material. Therefore, the RSM MXene‐hybrid anode material demonstrated remarkable rate capacity and superior long cycling life over 4000 cycles in a half‐cell. Whereas under a harsher condition, e.g., hydrothermal in NaOH solution, the Ti_3_C_2_T*_x_* MXene further underwent stepwise surface phase transformation. As reported by Sun *et al*.,^[^ ^136^ ^]^ RSM products consisting of NaTi_8_O_13_ and NaTiO_2_ phase (NTO) were detected on an NTO/Ti_3_C_2_ nanohybrid. By integrating the surface oxidation and post‐transformation of Ti_3_C_2_T*_x_*, Yang *et al*. recently constructed NASICON (sodium superionic conductor)‐type material of NaTi_2_(PO_4_)_3_ (NTP) on Ti_3_C_2_T*_x_* MXene to fabricate a dual‐mode hybrid anode for SIS.^[^ ^139^ ^]^ The NTP nanocubes were anchored on the nanosheets of MXene to realize an enlarged interlayer spacing for MXene, which benefited from the charge transfer (Figure 9D). Combining with the battery‐type behaviors from NTP and the pseudocapacitive characters of MXene, the dual‐mode MXene@NTP anode assembled in half‐cell device showed outstanding rate performance and excellent cycling stability over 10,000 cycles at 5 A g^−1^.

The performance of RSM MXene‐hybrids for SIS (including SIBs and SICs) is summarized in Table 2 and Table 3, respectively. It should be noted that although the SIS mechanism of either MXene or the aforementioned secondary phases has been illustrated in the past decades, the in‐depth understanding of the synergetic enhancement of SIS performance in RSM MXene‐hybrids was still a challenge. Therefore, a quantificational interpretation of the synergy of MXene and the RSM secondary phase awaits further exploration.

**TABLE 2:** The SIBs performance of the RSM MXene‐hybrids

RSM hybrids	Test condition (half/full‐cell,as anode/cathode, counter electrode)	Reversible capacity (mAh g^−1^@mA g^−1^)	Rate performance (mAh g^−1^@ mA g^−1^)	Cycling stability (current density, cycles, capacity retention)	Refs.
T‐MXene@C	Half‐cell, anode, Na plate	257.6@50	77.8@10,000	1000 mA g^−1^, 3000, 91.7%	^[^ ^127^ ^]^
Bulk O‐Ti_3_C_2_T*_x_*	Half‐cell, anode, Na plate	167@200	43@5000	1000 mA g^−1^, 2500, ∼76.5%	^[^ ^152^ ^]^
a‐Ti_3_C_2_ MNRs	Half‐cell, anode, Na plate	168@20	84@300	200 mA g^−1^, 500, ∼59.5%	^[^ ^138^ ^]^
TiO_2_/Ti_3_C_2_	Half‐cell, anode, Na plate	∼156@50	∼52@2000	200 mA g^−1^, 500, ∼100%	^[^ ^153^ ^]^
TiO_2_/Ti_3_C_2_	Half‐cell, anode, Na plate	237.8@100	151.5@1000	600 mA g^−1^, 100, ∼95.6%	^[^ ^132^ ^]^
a‐VO*_x_*/V_2_C	Half‐cell, cathode, Na plate	307@50	96@2000	2000 mA g^−1^, 1800, ∼56.2%	^[^ ^154^ ^]^
Na_0.23_TiO_2_/Ti_3_C_2_	Half‐cell, anode, Na plate	138@100	47@3000	2000 mA g^−1^, 4000, ∼100%	^[^ ^135^ ^]^
NTO/Ti_3_C_2_	Half‐cell, anode, Na plate	157@50	78@2000	/	^[^ ^136^ ^]^
MXene@NTP‐C	Half‐cell, anode, Na plate	208@100	102@10,000	1000 mA g^−1^, 2000, 74%	^[^ ^139^ ^]^

**TABLE 3:** The SICs performance of the RSM MXene‐hybrids

RSM hybrids	Test condition, counter electrode	Reversible capacitance	Rate performance	Cycling stability (current density, cycles, capacity retention)	**Refs**.
V‐MXene (Ti_3_C_2_T*_x_*)	Three‐electrode system, Pt	321.7 F g^−1^@10 mV s^−1^	/	/	^[^ ^150^ ^]^
CT‐S@Ti_3_C_2_‐450	Half/full‐cell, anode, Na plate/active carbon	550 mAh g^−1^@100 mA g^−1^ (half‐cell)	120 mAh g^−1^@15,000 mA g^−1^ (half‐cell)	2000 mA g^−1^, 10,000, 73.3% (full‐cell)	^[^ ^62^ ^]^
N‐Ti_3_C_2_T*_x_*	A single cell CDI device	514 F cm^−2^@2 mV s^−1^	∼290 F cm^−2^ @100 mV s^−1^	500 mA g^−1^, 2000, 99.75%	^[^ ^151^ ^]^

## 4 CONCLUSION AND PERSPECTIVES

In summary, sodium is shown to have a promising future as an alternative to lithium for next‐generation EESDs, while the low energy density and sluggish kinetics of current SIS materials remain unraveled. As the adjustable surface physicochemical property is closely related to sodium accommodation, the MXenes have shown great advantages on the SIS devices with high energy density and enhanced kinetics. In this review, the MXenes and their RSM derivatives for SIS applications are summarized. Particularly, the origin of the fast charge transfer, enhanced capacity, and facilitated reaction kinetics in MXenes and RSM MXene‐based hybrids have been considered in detail, which we believe would be beneficial for a comprehensive understanding of this new material system.

At the same time, it should be noticed that although plenty of works have been done to illustrate the underlying mechanism of SIS in MXenes and their RSM derivatives, most of them are based on theoretical predictions, while the evidence from laboratory observation is still rare. Particularly, since the interfacial coupling of RSM MXene‐based hybrids plays a critical role in the resulting Na‐ion adsorption, intercalation, and migration, further *in‐situ* techniques are necessary for monitoring the SIS behaviors during electrochemical processes. Additionally, most of the theoretical simulations are still limited to the simple interaction between Na atoms and the material so far, whereas the reactions at the interface of electrode/electrolyte are rather complicated. Therefore, with a decreased characteristic dimension and an enlarged specific surface area, more side reactions that are driven from the interface should be taken into consideration.

Furthermore, in consideration of the versatility of surface characteristics and species, the RSM MXene‐based hybrids are adequate for the host of cations beyond Na (e.g., K, Zn, Mg, or Al), and even anions (S_n_^2−^). Although these works are not included in the review, the advantages of MXene‐based hybrids on these aspects have already been revealed in recent years, which demonstrated the close relationship between the performance and the surface chemical states of the host materials. Therefore, a deeper insight into the surface‐dependent guest accommodation on RSM MXene‐based hybrids would be necessary for all these energy storage systems.

Last but not least, as aforementioned, the surface characteristics of MXenes can be controlled by varying synthetic routes, but usually less attention has been paid to alter the surface characteristics in the MXene‐based hybrids during synthesis, let alone an exclusive glance at the systematic summary of synthesis methods, e.g., the RSM. Upon the conflict between the complex reaction protocol for RSM MXene‐based hybrids and the goal of commercial SIS applications, in the future, more attention should be devoted to simplifying the reaction methods.

## CONFLICT OF INTEREST

Qingyu Yan is a member of the *Exploration* editorial board. The authors declare no conflict of interest.
